# A proposed workflow for proactive virus surveillance and prediction of variants for vaccine design

**DOI:** 10.1371/journal.pcbi.1009624

**Published:** 2021-12-16

**Authors:** Jordan J. Baker, Christopher J. P. Mathy, Julia Schaletzky

**Affiliations:** 1 Joint Graduate Program in Bioengineering, University of California, Berkeley and University of California, San Francisco, Berkeley, California, United States of America; 2 Innovative Genomics Institute, University of California, Berkeley, Berkeley, California, United States of America; 3 Center for Emerging and Neglected Diseases, Immunotherapy and Vaccine Research Initiative, University of California, Berkeley, Berkeley, California, United States of America; University of Virginia, UNITED STATES

## Introduction

Resolving the Coronavirus Disease 2019 (COVID-19) pandemic is hamstrung by emerging Severe Acute Respiratory Syndrome Coronavirus 2 (SARS-CoV-2) variants with the potential to evade both natural and vaccine-induced immunity. Variants occur naturally from replication errors by viral polymerases and escape variants arise when virus mutations allow evasion of recognition by the immune system or therapeutic treatment. The selective pressure of vaccines, innate immune responses, and clinical interventions fosters the emergence of mutations that confer the capacity to continue infecting new hosts [1]. These variants of concern (VOCs) add complexity to public health responses when they are more transmissible, deadlier, or when they decrease the efficacy of vaccines and treatments. These variants also stoke public uncertainty and fear as well as increase vaccine hesitancy. While SARS-CoV-2 variants have dominated the news recently, variants have driven more transmissive versions of Ebola [2], affected vector tropism for the chikungunya virus [3], and made many other viruses more likely to cause pandemics.

Rapid characterization of variants for future viruses is therefore essential to an effective pandemic response. If we can predict and characterize VOCs before they arise, monoclonal antibodies and potential vaccine antigens could be developed to proactively neutralize these variants. Furthermore, better understanding of the molecular characteristics of each variant could guide more precise public health policies.

Currently, variants are identified and functionally tested using a combination of viral sequencing, cell-based assays, protein engineering methods, and computational tools. Sequencing of viral genomes from infected hosts identifies mutations away from the earliest reported genome of the virus, called the reference genome, highlighting possible variants. After identifying these mutations, cell and protein-based assays are used in the lab to test whether these mutations enable escape from neutralizing antibodies, which are antibodies created by the immune system from previous infection or vaccination [4,5]. Mutations of interest are generated in a virus or pseudovirus and convalescent plasma containing neutralizing antibodies from people previously infected or vaccinated are tested for efficacy of neutralization [6,7].

As methods for variant characterization become increasingly powerful, we have yet to see effective coordination of the research groups conducting these studies across disciplines. Although the United States spends billions of dollars annually on infectious disease research and development, including genomic surveillance for identifying variants [8], this investment has resulted in remarkably poor results for genomic surveillance. The Centers for Disease Control and Prevention (CDC) recently upgraded their surveillance system in January of 2021 but is still only able to accommodate sequencing of 750 viral samples per week through their NS3 system [9]. While the US has one of the highest capacities for sequencing between the private and academic sectors, the rate of sequencing ranks 33rd in the world with a rate of sequencing at less than 2% of total cases, well below the necessary mark for identifying variants early [10,11]. One cause of this poor response comes from the complicated interactions between diagnostic labs/clinics and the facilities performing sequencing. Health Insurance Portability and Accountability Act (HIPAA) and Institutional Review Board (IRB) approvals along with patient consent must be obtained. Additionally, the cost of the sequencing needs to be funded by someone. These complications slowed sequencing and therefore identification of VOCs.

In preparation for future pandemics, we propose establishing a consortium of research groups with expertise in both computational and experimental techniques to more exhaustively map the landscape of variants upon the emergence of a new virus and predict which variants are most likely to arise. This would enable a rigorous set of standard analyses for assessing potential VOCs, as well as allow for early design of vaccines with efficacy against the variants deemed most likely to arise. While this work is currently being done for SARS-CoV-2 at multiple universities, companies, and government agencies, we can use lessons from this pandemic to propose a more rapid and coordinated response for future emerging viruses. Below, we highlight recent technological advances in these fields. By combining existing technology and new technologies, we envision a collaboration of groups employing computational and experimental tools to quickly predict and characterize likely VOCs, which could be used to aid initial identification of neutralizing monoclonal antibodies and vaccine design when a new virus emerges and inform policy decisions as variants arise.

## Proposed workflow to predict variants of concern

An efficient approach to predict viral variants would be for the US to invest in a new system for predicting and testing likely VOCs immediately upon identification of a new virus. This workflow would be handled by experts in the individual technologies working collaboratively and sharing data in real time. While we propose technologies that already exist, they are implemented in different labs around the world with different levels of expertise, requiring communication, collaboration, and sharing of information. One delay in the US response is bringing together these experts, allowing access to clinical surveillance samples and coordinating efforts between labs in a meaningful manner. The current variant response approach in the US is reactive since these collaborations take time to develop after a new virus becomes a concern to public health, increase the administrative burden for hospitals and diagnostic laboratories who are already overstretched in a pandemic and have to ensure compliant sample handling and patient consent to surveillance studies, and could ultimately cost thousands of lives, billions of dollars, and extend a pandemic because the nascent collaboration is not fast enough to prevent the spread of new variants.

**Fig 1:**
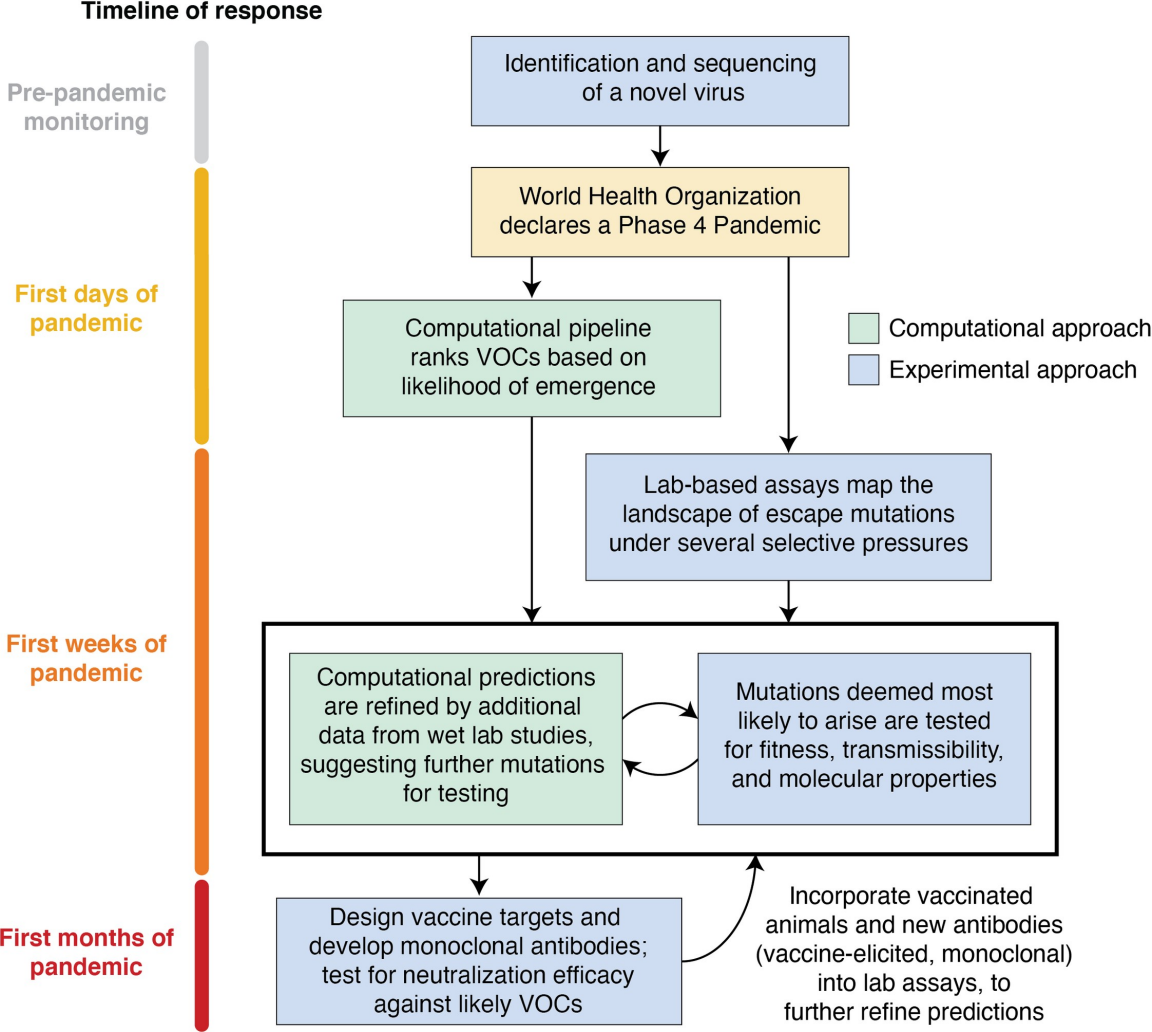
Proposed workflow of consortium to predict and characterize viral VOCs. Starting with an input of a viral sequence, computational tools and lab-based assays can be implemented to predict VOCs. The results from these approaches, run by experts on the methods, can be fed back into each other to further refine predictions. VOC, variant of concern.

Instead, this proposed workflow, described in Fig 1 and below, would take a viral genome, as soon as it is first sequenced, as an initial input to the computational models to predict likely VOCs. Below, we discuss several technological approaches that seek to define regions of the genome where mutations are likely to occur and identify specific point mutations that would likely create VOCs, similar to how flu vaccines are predicted each year [12]. Another output would be regions of the virus that are likely to remain stable and would be ideal epitope targets for vaccines. These predictions would be fed into and complemented by lab-based technologies to evaluate candidate VOCs, provide additional data for generating improved predictions, and ultimately identify optimal viral epitopes for vaccine targets as continued rounds of computational prediction and experimental verification will support higher certainty around which variants are of most concern. Combining expertise and technologies into one consortium with predetermined methods for sharing data, collaborating, and working efficiently when called upon will minimize delays between groups and allow efficient sharing of samples. The consortium would conduct routine viral surveillance for influenza and other seasonal viruses during nonpandemic times, establishing workflows and shared resources, maintaining active collaboration, and improving and testing computational prediction models. By rapidly expanding this coordinated workflow immediately upon the World Health Organization (WHO) declaring a virus reached Phase IV of the pandemic scale (triggered by evidence of sustained human to human transmission), initial vaccines and therapeutics can be designed with maximal efficiency against predictable VOCs, saving lives and shortening pandemics.

## Current technologies for assessing variants

### Cutting-edge computational tools

Computational approaches can rapidly identify VOCs early in a pandemic, especially given the reduced need for time, resources, and safety regulations associated with many computational approaches. Computational tools are a necessity since there are approximately 20^n single coding mutations, let alone multiple mutations and indels. While experimental tools are still the gold standard, the best experimental approaches, including pooled screening experiments, would not be able to handle every possible mutation.

We envision this coordinated response would be activated upon obtaining the genetic sequence for a new virus. With only the genome, computational tools such as BLAST [13] and other well documented algorithms and models can be applied to predict identity, structure, and function of proteins from the genome [13–16], including those developed explicitly for annotating single viral genomes [17,18]. From there, other computational tools could predict regions that would likely change in VOCs.

One notable recent study implemented a deep learning algorithm known as a language model to build a powerful predictor of variants [19]. The model, trained on protein sequences from different viruses, relies on the principle that viruses within the same family use a common set of proteins (encoded in their genome) to replicate. By learning the sequence patterns of proteins for other viruses in a family, the model can assess whether a new genome is likely to encode functional viral proteins. These viral proteins could then be fed into a language model like the one described by Hie and colleagues [19]. This type of model was first built to learn the grammars of languages—for example, learning the syntax of the English language and being able to classify whether a new sentence is grammatically correct. The authors utilized the large collection of previously sequenced genomes for viral families to train their predictor, which showed impressive performance in distinguishing known escape mutations from the set of all mutations seen in a virus. The approach is generalizable, successfully identifying known VOCs for multiple viral families. Importantly, training the model did not require explicit data on what variants exist and were VOCs, but relied on large amounts of sequencing data from other viruses, making it an ideal first step for mapping the mutation landscape early in a pandemic, before genomic surveillance has provided evidence of mutations.

Another promising computational technique for early interrogation and prediction of viral changes are models based on nucleotide composition. It is known that nucleotide and dinucleotide compositions of viruses evolve to match their host species to evade detection and subsequent clearance by cellular defense systems [20,21]. Accordingly, computational models have been developed that use quantification of viral genome nucleotide compositions to predict sequence changes in viral strains as they adapt to humans from other animal hosts [22,23]. These methods were successful in retroactively identifying adaptive sequence changes most likely to occur for influenza. Thus, training a model for a newly emerged pathogen to score new mutations based on their change to nucleotide composition is an appealing possible approach for characterizing the likelihood of emergence of new variants. Importantly, the composition of dinucleotides has been shown to be a property of virus family more so than host species [20], suggesting that training a computational model on viral sequences identified from nonhuman hosts could be informative in the early days of a pandemic, before the virus has had time to spread and sequences identified from hosts are sparse and highly similar. However, these models are limited in that they do not incorporate any molecular or structural details into which mutations are more or less likely to arise. Mutations at different sites in the genome that similarly increase or decrease the composition of a given nucleotide pattern would be scored identically. These models would therefore best be used in combination with other computational approaches to refine rankings of VOCs.

Computational modeling of the structural effects of mutations is another approach that provides rapid molecular characterization of a large set of viral variants. For example, molecular dynamics (MD) simulations have characterized the change in binding affinity of the SARS-CoV-2 spike receptor-binding domain to the human entry receptor angiotensin converting enzyme 2 (ACE2) upon mutation [24], and in one case, the ensemble of energies calculated by the MD trajectories was used to train a neural network to predict the apparent K_*D*_ of interaction [25], achieving correct variant classification of >80% for a blind test set of 54 variants. Structures of the spike protein [26] and the spike–ACE2 complex [27] were available remarkably early in the pandemic, with structure coordinates being deposited in the Protein Data Bank for public access on February 10, 2020 and February 21, 2020, respectively, less than 3 weeks after WHO declaration of a public health emergency on January 30, 2020. Thus, computational protein modeling techniques are readily accessible tools for use early in the pandemic for predicting the molecular details of variants that may increase transmissibility or disease severity. Determining which molecular details are most relevant for predicting VOCs may be highly dependent on the virus of interest, underlining the benefit of following up on computational modeling predictions with experimental studies.

As the pandemic progresses and novel variants arise, their genomes will be collected into databases [28] and fed into these computational models as additional training data to improve their accuracy and aid design of booster vaccines if necessary. In the later stages of the pandemic when many strains have been collected, the accumulation of sequences will also permit additional computational techniques for predicting the long-term evolution of a virus. For example, computing the Shannon entropy for each amino acid position of the spike protein across a large number of sequences (>310,000) from GISAID’s EpiCoV database [29] enabled one group to identify mutational hotspots, including many positions at which mutations had already occurred in VOCs [30]. Of note, the L452R and E484K mutations that were recognized in VOCs in 2021 lie in regions that the model identified as hotspots despite only using data from 2020. This study highlights the ability of a large number of sequences to provide information on future evolutionary trajectories of viruses. Lastly, if a viral strain becomes endemic, fitness models can be used to predict the evolution of different clades of a virus each year, as is done for influenza [31].

In sum, these computational methods provide an enormous opportunity to support efforts to predict and identify VOCs, traditionally driven by experimental techniques. For example, finding suitable animal models posed an initial challenge for studying SARS-CoV-2 because it was unable to infect mice due to differences in the ACE2 receptor protein used for viral entry. Computational techniques allow for molecular insight independent of the access to these and other resource-intensive experimental techniques. Furthermore, computational approaches offer the unique opportunity to integrate information on the virus sourced from many different types of analyses, such as incorporating observed VOC frequencies from genomic surveillance to refine the predictions made by the models, thus accounting for human-specific factors that are not captured by the molecular mechanisms that form the basis of the computational techniques. Importantly, incorporating continuous low-level surveillance in the proposed consortium model will allow continuous refinement of computational methods, across several families of viruses that occur seasonally. This could also help better model seasonal influenza strain development.

### Lab-based experimental tools

Complementing the computational tools, lab-based technologies could simultaneously predict variants and feed additional information to the computational tools. The most straightforward approach to mapping the mutational landscape is to simply test each one in turn, using a technique known as deep mutational scanning or saturation mutagenesis. In a series of studies, the group of Dr. Jesse Bloom at the University of Washington used an exhaustive library of single nucleotide variants of the SARS-CoV-2 spike protein receptor-binding domain expressed on the surface of yeast to test for binding to the entry receptor ACE2 [32], for escape from therapeutic antibodies [33,34], and for escape from neutralizing monoclonal [35] and polyclonal [36] antibodies derived from SARS-CoV-2 convalescent patients. This approach has also previously been used to predict fitness for human influenza variants [37]. These studies showcase the benefits of mutation mapping using saturation mutagenesis, namely that the exhaustive library can be rescreened for measurement of several different properties. The results from the various screens can be integrated into a more complete description of the molecular properties of each potential VOC, which may inform therapeutic strategies.

To map the escape landscape of a whole viral genome, laboratory researchers incubate the virus with cells and potential treatments to examine how the virus mutates to adapt to selective pressures [5]. This approach has been used to identify mutations in HIV [38], influenza [39], and for escape variants of SARS-CoV-2 elicited under the selective pressure of remdesivir, a widely used therapeutic targeting the RNA-directed RNA polymerase of SARS-CoV-2 [40]. By screening for increased viral fitness in the presence of remdesivir challenge, the authors identify mutations to the RNA polymerase and, surprisingly, mutations in the spike protein. These known spike protein mutations as well as those in the exonuclease nsp12 are rare, but have been observed in the broader population and could arise without stringent selective pressure. This selection can additionally be performed using panels of common drugs and treatments for a wide range of viruses [41]. Proactively mapping the escape landscape under the selective conditions of multiple therapeutics and combination of therapeutics used worldwide not only helps us better predict viral evolution but may afford us crucial time for designing new drugs with improved potency against variants before they become widespread.

Lab-based assays can also be performed to identify epitopes for vaccine targets that would be less prone to mutations by finding epitopes and antibodies that have high binding affinities across related viral species. Recently, Wang and colleagues identified the main antigens of the SARS-CoV-2 spike (S) protein targeted by the immune system, which were distinct from antigens that are more conserved between SARS-CoV-2 and SARS-CoV-1 [42]. By eliminating the main epitopes in a lab strain of the virus and infecting mice, the mouse immune cells produced antibodies that targeted the conserved antigens and were more broadly neutralizing against both SARS-CoV species. Additional lab-based assays find that antibodies targeting more conserved regions help to elicit cross-reactive neutralizing antibodies efficacious against variants, providing excellent information on what epitopes to use as the basis for a vaccine [4,6,43]. While some of the conserved epitopes could induce weaker immune responses, using these techniques to identify conserved epitopes that elicit strong responses will be critical for designing durable efficacious vaccines. These types of studies identify those domains of viral proteins most likely to develop variants, as well as which regions are more stable and therefore could be better targets for vaccines and therapeutics.

## Implementation and safety considerations

To implement this system, strong computational expertise, tools, and access to computing power are needed within the consortium so that results can be quickly utilized by all assays to predict variants and vaccine candidates. The consortium would be active at a “maintenance” level continuously, characterizing the yearly influenza pandemic as well as several cold virus strains, conducting surveillance and optimizing training algorithms on the emergence of variants. This could improve the influenza vaccine selection and our response to other seasonal pathogens. We could also learn a lot about virus surveillance, transmission and mutagenic behavior of viruses, potentially informing public health recommendations for nonpandemic viruses that still cause significant morbidity (i.e., common cold coronaviruses). A consortium conducting continuous low-level virus surveillance in the population could easily ramp up activities when a virus reaches Phase IV of WHO’s pandemic phase designation, which includes sustained human to human transmission. The sequence of this virus will be immediately fed into the computational models above (by the labs in the consortium with expertise in the computational models) while simultaneously being used in the lab-based assays (by other labs in the consortium with appropriate biosafety labs and expertise), with results being shared automatically among all members. These results will be fed into a central database maintained by the consortium and distributed to each member to update their respective work. Ultimately, a list of most likely variants and potential phenotypes associated (i.e., more transmissible, more severe disease, etc.) will be output along with potential vaccine and therapeutic targets within a few weeks to months.

There are examples of many successful consortia, including The Broad Institute’s Interdisciplinary Research Consortium, the Enzyme Function Initiative funded by the National Institute of General Medical Sciences, and many others. The Viral Hemorrhagic Fever Consortium (VHFC) is a great example of a nonprofit consortium starting from an initial grant, spanning multiple universities, countries, and agencies and has since expanded to include many additional partners. The majority of existing consortia focus on a single virus or group of viruses, while this consortium could be expanded to include a broad range of experts spanning all viral families. Using lessons learned from these successful consortia as well as best practices for governance, reward structures, and implementation of consortia [44,45], we believe this consortium can be supported sufficiently with funding from an organization such as the National Institutes of Health (NIH) or CDC. Labs with varying expertise to cover the computational and experimental methods can be identified by the funding organization and invited to join, with appropriate governance and reward structures to allow members to still publish results. Splitting the consortium among different labs and organizations allows the best experts across different fields collaborate while minimizing the burden placed on any single lab. Importantly, a focus on computational methods would allow immediate results that could be used for vaccine antigen selection—for cell-based studies, a virus sample has to be obtained together with the required safety approvals, and then a permissive cell line needs to be found and an animal model established. This can take significant time and delay the generation of data needed to develop optimal vaccines and therapeutics. As sequencing does not require culture of the virus, sequence data can be obtained directly from clinical samples. Within the proposed consortium, initial predicted algorithms could be run within hours after a sequence is available, allowing rapid response times and a more informed choice of vaccine antigen, for example.

The ability to centralize consent forms, IRBs, and other documentation while gaining access to large data sets will entice many labs to join this consortium. This consortium would be formed with NIH support, which could help develop “blanket” IRBs and consent forms to increase sequencing of samples throughout future pandemics by clinical and diagnostic labs. By working with these labs, the consortium could facilitate increased sequencing, improving surveillance for VOCs while providing additional data for the computational models and increasing efficacy of the consortium. These computational methods were applied to SARS-CoV-2 even at the poor sequencing rates in the country, so the additional sequencing would not be critical, but would certainly help policy makers and the consortium.

There are many documented benefits to collaborative research, and this consortium would improve access to different resources, increase professional networks, minimize burdens on individual labs, provide additional funding for labs, provide opportunities for publishing, and ultimately speed up research that has the potential to impact the entire world. However, the consortium needs to be managed and funded well, be ready to act when called upon, and established structures and workflows (i.e., access to clinical surveillance samples) need to be sufficiently robust to support a rapid expansion of activities in case of a pandemic. Best practices mentioned above and a significant amount of learnings from the COVID-19 pandemic can help implement a system that helps prepare for and mitigate future pandemics.

Additionally, virology and basic biology expertise is required, especially related to the lab-based assays working with high containment pathogens at Biosafety Level 3. The verification experiments we propose would comprise in vivo lab technologies requiring a Biosafety Level 3 and use mainly pseudovirus assays to avoid working with dangerous live viruses. It is of utmost importance that more virulent virus strains are only generated using precautions such as pseudovirions and appropriate containment and that once the computational tools are trained to be highly predictive, safety measures against abuse are taken, and both details of the tools themselves and sequences of highly virulent proposed viruses are appropriately safeguarded together with NIH and other government institutions. However, if the consortium is successful in predicting early variants that are moderately efficient at evading selective pressure and this informs improved vaccine design with future variants in mind, the risk of developing a highly virulent strain is in practice significantly reduced. Allowing a virus to “fester” with only partially effective vaccines over long periods of time will increase risk for more virulent variants.

Another important consideration for VOC prediction is the different selective pressures experienced by the virus in parallel as it infects different hosts. For example, variants of SARS-CoV-2 are thought to have arisen in farmed minks before transmitting back to humans in Denmark [46] and Poland [47]. Despite arising in a nonhuman reservoir, the spike protein receptor-binding domain mutation Y453F from the Denmark variant showed enhanced binding to the human ACE2 receptor, which mediates SARS-CoV-2 entry into cells and may have increased its transmission potential among humans. These types of mutations have been seen for many viruses before SARS-CoV-2, including the chikungunya virus [3], influenza [48], and others. While most of the computational tools do not specifically include host species differences, many of them are agnostic to the host they arise in and mainly focus on the effects in humans. Genetic heterogeneity within human communities is an additional confounding factor as host genetic factors have been shown to have an impact on vaccine efficacy [49,50]. As initial vaccine rollout progresses, areas with high incidence of infection among vaccinated people due to decreased vaccine efficacy would result in viral selection under a partially effective immune response. A similar effect may occur in reinfected individuals, who may have a waning immune response. Communities with significant numbers of these types of infections are likely to generate VOCs and thus should undergo increased genomic surveillance.

Additionally, computational modeling and prediction efforts separate from the broader workflow may be warranted: While the computational models described above showed success in separating viral sequences from different host species for both influenza viruses and coronaviruses [14], the ability to actively predict how the presence of heterogeneous reservoirs influences viral evolution and variant selection globally is still a challenge. Incorporating animal data in the yearly surveillance studies proposed under this consortium could help model virus transmission also in animals living in close proximity to humans.

## Conclusions

The vision we present for an ideal response to the identification and sequencing of a new virus is to maintain at a low level and in case of pandemic, rapidly deploy both a computational and a lab-based assay pipeline to predict and characterize the most likely VOCs, and define optimal monoclonal antibody and vaccine targets. While many of these technologies were created for previous viruses and pandemics, they have been updated and now include many more computational aspects because of SARS-CoV-2. We have highlighted many older and newer techniques that would benefit from integration in a consortium of experts that could be activated at a specific time after new a virus is identified. This coordinated approach would combine the power and speed of computational methods with the accuracy and translation of lab-based assays. By preemptively organizing this group around recurring influenza and other seasonal virus outbreaks, algorithms could be trained and workflows optimized for continuous low-level virus surveillance, allowing a rapid and efficient ramp-up during outbreaks that could become pandemics. A coordinated effort could predict VOCs early to aid vaccine design and guide public health policies. These candidate vaccines would have a greater likelihood of being able to prevent and treat variants that arise or even prevent variants from arising by squashing transmission early in a pandemic. This approach could also be used to design booster shots for a pandemic even after initial vaccines are available. Additionally, the characterization of each viral variant can also provide critical data to guide public health messaging as new variants do begin circulation. This broadly applicable workflow would ultimately cost a fraction of what the US is spending on infectious disease work in addition to saving lives during future outbreaks and generate unprecedented insight into virus transmission and evolution.
